# Quantitative visualization of subcellular lignocellulose revealing the mechanism of alkali pretreatment to promote methane production of rice straw

**DOI:** 10.1186/s13068-020-1648-8

**Published:** 2020-01-17

**Authors:** Xiaoli Li, Junjing Sha, Yihua Xia, Kuichuan Sheng, Yufei Liu, Yong He

**Affiliations:** 1 College of Biosystems Engineering and Food Science, Zhejiang University, 866 Yuhangtang Road, Hangzhou, 310058 China; 2 Key Laboratory of Spectroscopy Sensing, Ministry of Agriculture and Rural Areas, 866 Yuhangtang Road, Hangzhou, 310058 China; 3 State Key Laboratory of Modern Optical Instrumentation, College of Optical Science and Engineering, Zhejiang University, 866 Yuhangtang Road, Hangzhou, 310058 China

**Keywords:** Raman, Chemical imaging, Lignocellulosic biomass, Alkali pretreatment, Rice straw, Spectral unmixing

## Abstract

**Background:**

As a renewable carbon source, biomass energy not only helps in resolving the management problems of lignocellulosic wastes, but also helps to alleviate the global climate change by controlling environmental pollution raised by their generation on a large scale. However, the bottleneck problem of extensive production of biofuels lies in the filamentous crystal structure of cellulose and the embedded connection with lignin in biomass that leads to poor accessibility, weak degradation and digestion by microorganisms. Some pretreatment methods have shown significant improvement of methane yield and production rate, but the promotion mechanism has not been thoroughly studied. Revealing the temporal and spatial effects of pretreatment on lignocellulose will greatly help deepen our understanding of the optimization mechanism of pretreatment, and promote efficient utilization of lignocellulosic biomass. Here, we propose an approach for qualitative, quantitative, and location analysis of subcellular lignocellulosic changes induced by alkali treatment based on label-free Raman microspectroscopy combined with chemometrics.

**Results:**

Firstly, the variations of rice straw induced by alkali treatment were characterized by the Raman spectra, and the Raman fingerprint characteristics for classification of rice straw were captured. Then, a label-free Raman chemical imaging strategy was executed to obtain subcellular distribution of the lignocellulose, in the strategy a serious interference of plant tissues’ fluorescence background was effectively removed. Finally, the effects of alkali pretreatment on the subcellular spatial distribution of lignocellulose in different types of cells were discovered.

**Conclusions:**

The results demonstrated the mechanism of alkali treatment that promotes methane production in rice straw through anaerobic digestion by means of a systemic study of the evidence from the macroscopic measurement and Raman microscopic quantitative and localization two-angle views. Raman chemical imaging combined with chemometrics could nondestructively realize qualitative, quantitative, and location analysis of the lignocellulose of rice straw at a subcellular level in a label-free way, which was beneficial to optimize pretreatment for the improvement of biomass conversion efficiency and promote extensive utilization of biofuel.

## Introduction

Biomass energy, as a renewable carbon source, has attracted a lot of research interest in response to the energy crisis of recent years. It can, not only, help for resolving the management problems of lignocellulosic wastes, but also helps to alleviate the global climate change by controlling environmental pollution raised by their generation on large scale [1–3]. The bottleneck problem of extensive utilization of many biofuels lies in the intrinsically refractory nature of biomass, the filamentous crystal structure of cellulose, and the embedded connection with lignin in biomass such as in the straw cell wall that that leads to poor accessibility, weak degradation and digestion by microorganisms. This results in inefficient conversion of biomass [1, 4, 5]. To make lignocellulosic biomass less refractory, chemical pretreatments (the application of strong acids or bases), physical pretreatments such as ball milling [6], and other methods such as thermal and biological (application of microorganisms to decompose lignin and lignocellulose) pretreatments have been extensively studied and applied in recent years. Some pretreatment methods showed a significant improvement of gas yield and production rate [4, 7–11].

However, the promotion mechanism of pretreatment, especially the effect on lignocellulose in different types of cells in biomass tissues, has not been thoroughly studied. There is a typical heterogeneous structure of biomass material, in which there are approximately 35 cell types, with distinctive shapes, sizes, locations, and cell wall characteristics. Revealing the temporal and spatial effects of pretreatment on lignocellulose in diversified cells of heterogeneous biomass tissue will thus greatly help deepen our understanding of the optimization mechanism of pretreatment, and promote efficient utilization of lignocellulosic biomass.

The development of micro-spectrometers, which fuse the chemical specificity of vibrational spectrums with the power of optical magnification by the acquirement of high-quality spectra with subcellular resolution, has also contributed to further exploitation and utilization of biomass [2]. Microscopy based on infrared absorption offers chemical specificity, but the spatial resolution is limited by long infrared wavelengths, and the penetration depth into aqueous plant samples is limited [12, 13]. Raman microspectroscopy with advantages such as label-free chemical contrast, high spatial resolution, and chemical specificity, which are free from water disturbance, has been widely used in visualizations of subcellular lignocellulose [14]. Segmehl et al. adopted Raman spectroscopy to image the spatial alternation of lignin in CAD deficient transgenic poplar during delignification [15]. Toru Kanbayashi et al. used Raman microscopy to reveal the lignocellulose variation of wood under artificial weathering [16]. Ji et al. illustrated the distribution of lignin and cellulose in wood based on confocal Raman microscopy [17]. Foston et al. used coherent anti-Stokes Raman scattering (CARS) to image lignin distribution in cross sections of tension wood [18]. Sarr et al. adopted stimulated Raman scattering (SRS) microscopy to study the spatiotemporal variation of lignin and cellulose in the transection of core stover stems during the degradation of biomass [19]. Richter et al. used the Raman spectroscopy to image lignin, cellulose, and pectin distributions and conduct semi-quantification analyses [20]. The above results indicate that Raman spectra have been successfully used to analyze the changes of lignocellulose in biomass. However, lignocellulosic compositional distribution by mapping of integrated areas or intensities at a diagnostic spectral band of the compound in the current researches may be affected by severe disturbance. Raman spectrum is a typical weak signal, its probability of occurrence is about one in ten million compared to Rayleigh scattering [14], and is especially susceptible to background fluorescence interference of plant tissues, which makes it difficult to achieve reliable quantitative and location analysis based on single-band Raman intensity [21]. So, single-band Raman spectral image often resulted in an obvious deviation from macroscopic quantitative measurement results, and highly similar distribution maps of different lignocellulosic compositions.

Therefore, more reliable Raman chemical imaging methods should be developed to deepen understanding of the reactions of pretreatment to subcellular lignocellulose. In this study, we proposed a hybrid of the confocal Raman microspectroscopy technique and the spectral unmixing algorithm with full-range spectral constraints to illustrate the temporal and spatial variation of lignocellulose induced by alkali pretreatment. The main goals of this study were to: (1) nondestructively explore the difference in cell wall structure or morphology of rice straw before and after alkali treatment based on the Raman fingerprint spectral and statistical analysis; (2) establish a label-free Raman chemical imaging approach based on spectral unmixing with a full-range of spectral constraints of lignocellulosic reference standards; (3) visualize and quantify the temporal and spatial variation of subcellular lignocellulose in the transection of rice straw during alkali treatment in a label-free way.

## Materials and methods

### Anaerobic fermentation experiment

#### Raw substrate and inoculum

Rice straw was collected from the Changxing Agricultural Science and Technology Park experimental farm of Zhejiang University in October 2013. After natural air-drying the straw was cut to 2–4 cm and then stored in PE plastic bags to be reserved. The straw’s total solid content (TS) was 89.8% (wet base, wb) and volatile solids content (VS) was 77.9% (wb). Anaerobic fermented inoculated sludge was taken from the Zhengxing biogas plant in Hangzhou (pig manure was used as fermentation material) and the TS of the inoculated sludge was 15.4% (wb), while VS was 7.8% (wb), and the pH value was 7.6.

#### Sodium hydroxide pretreatment

A total of 6 g of sodium hydroxide (with a purity level of 97%), 1300 ml distilled water, and 100 g chopped straw were added to a 2000-ml beaker. It was then put in a water bath at 100 °C for 1 h. Then, the pH was adjusted to neutral with a hydrochloric acid solution. After pretreatment with sodium hydroxide, the TS of the rice straw was 15.6% (wb) and VS was 12.8% (wb).

#### Anaerobic fermentation

Batch anaerobic fermentation was carried out for rice straw pretreated with sodium hydroxide, and for straw without pretreatment, respectively. Rice straw with a total solid content of 27 and 180 g of inoculated sludge were added to a 1000-ml anaerobic fermentation flask, and an appropriate amount of carbamide was added to adjust the C/N ratio to 25. A certain amount of purified water was added to the rice straw in the untreated group, making the moisture content of the rice straw consistent with that of the sodium hydroxide treated group. The total volume of the mixture in the bottle was 300 ml. After connecting the fermentation device, argon gas was injected into the device to remove air, and then fermentation flask was placed in a water bath cauldron at a temperature of 35 ± 1 °C. Biogas was collected by discharging saturated salt water (adjusting salt water with sulfuric acid to make its pH < 3) and stored in a 1000-ml glass storage cylinder. The daily biogas production was calculated and converted into the gas volume under standard conditions (0 °C, 101 kPa) according to the temperature and pressure at that time.

#### Measuring indices and methods

The gas composition of biogas was detected by a gas chromatograph every 5 days. The conditions of the gas chromatograph (Shimadzu-GC 2014, Japan) were as follows: the temperature of the TCD detector and packed chromatographic column were 80 °C and 60 °C, respectively. Argon was used as a carrier gas and the flow rate was 30 ml/min. Column box, sample inlet, and heat conduction detector temperatures were 100 °C, 120 °C and 120 °C, respectively. The injection volume was 200 μl. The measurement of TS (dried in an oven at 105 ± 1 °C for 24 h) and VS (mass loss at 600 ± 2 °C for 2 h) of chopped rice straw was based on the 2540G standard method [22]. The concentration of hemicellulose, cellulose, and lignin were measured according to the Van Soest method [23]. Before measurement, all the samples were dried and ground into powder with a diameter of less than 400 microns.

### Microscopic sample preparation

Sample blocks of 1 × 2 cm^2^ were cut off from untreated and alkali-treated rice straw and fixed in a 2.5% glutaraldehyde solution at 4 °C. After conventional treatment, Spurr resin was embedded [24]. Then, semi-thin transverse sections with a thickness of 2 μm were cut from the embedding block by a microtome (11800 Pyramitome, Sweden) for Raman micro-spectroscopic acquisition.

In terms of electron microscopic observation, the samples were sliced in an ultramicrotome (Reichert, Germany), and 70–90 nm sections were obtained. The sections were stained with a lead citrate solution and a 50% ethanol-saturated solution of uranyl acetate for 15 min, and then observed in a Hitachi H-7650 transmission electron microscope (Japan) [25].

### Raman spectrum acquisition

Raman spectrum was collected using a Renishaw confocal Raman spectrometer (Renishaw Plc., Wotton-Under-Edge, UK) with a focused Nd:Yag laser (*λ* = 532 nm). The prepared sample slices were fixed on the object stage under a 50 × objective lens, and the Raman micro-spectroscopy of transection was acquired using the point-by-point scanning mode in the spectral range of 580–3062 cm^−1^. The exposure time and the laser intensity were 50 s and 0.5 mv, respectively, the experiment was carried out at ambient temperature of 25 °C.

Spot scanning: a total of 574 samples, including 235 untreated and 339 alkali-treated samples, were obtained. Each sample was generated by averaging 25 spectra acquired from a microscopic region with a size of about 10 × 10 μm, and these microscopic regions covered various types of cells in cross-sectional tissues of the rice straw, including epidermis, chlorenchyma, collenchyma, parenchyma, phloem, sclerenchyma, spongy parenchyma, vascular bundle, and xylem.

**Fig. 1:**
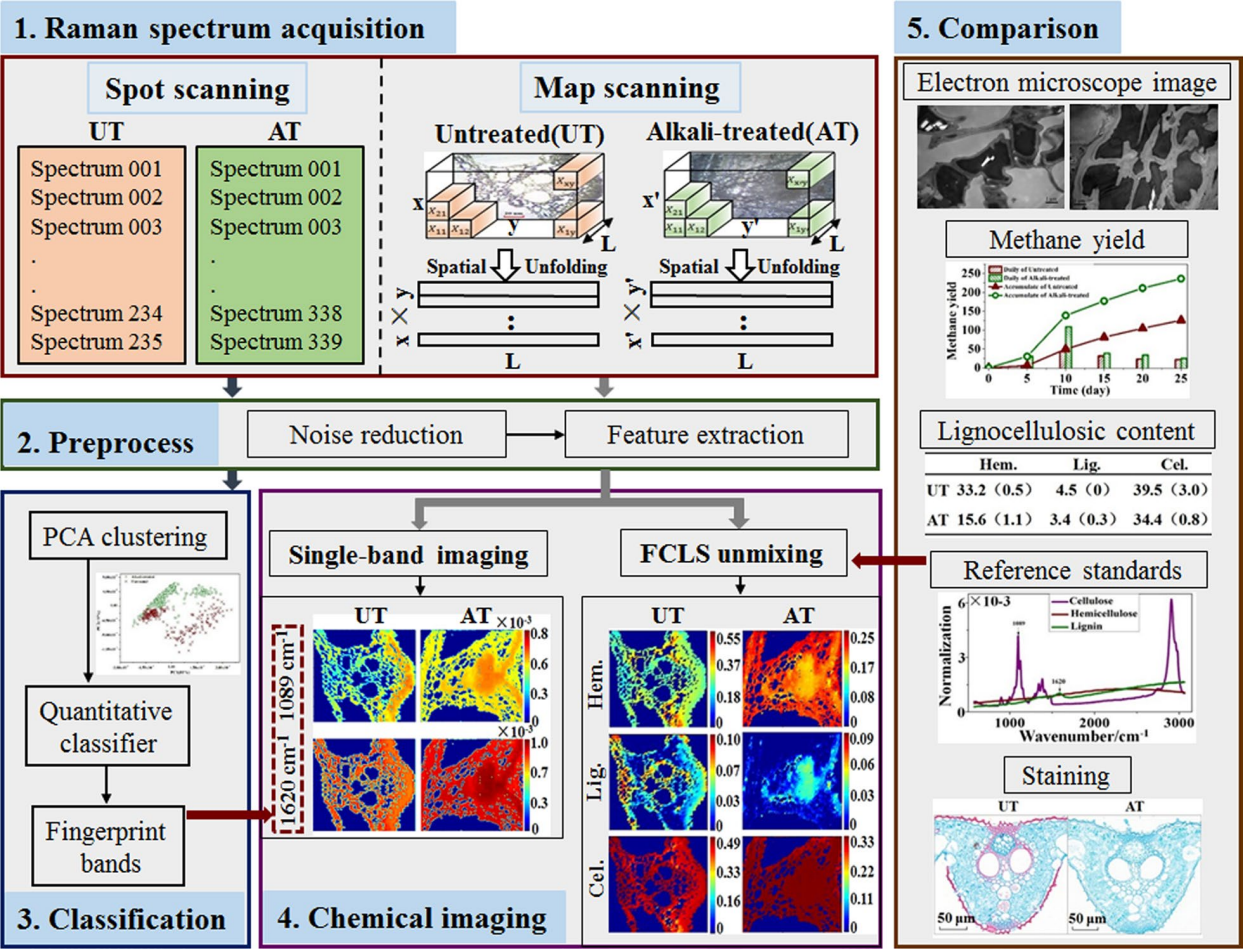
System framework diagram of this study

Map scanning: four randomly selected transections were taken for Raman micro-spectra mapping, two for each treatment. For the alkali-treated transection, two mapping scanning images were, respectively, obtained, one with 1950 spectra and 39 × 50 pixels, and another with 11,495 spectra and 95 × 121 pixels. For untreated samples, the two mapping scanning images were obtained with 3685 spectra and 55 × 67 pixels, and with 4992 spectra and 78 × 64 pixels acquisition, respectively. The sampling spatial resolution was 2.5 × 2.5 μm. The mapping area is illustrated in “Raman spectrum acquisition” in Fig. 1.

To better understand the effects of alkali treatment on the spatial and temporal distribution of lignocellulosic components in straw, three lignocellulosic compounds of lignin (CAS: 8068-05-1, Sigma Aldrich), cellulose (CAS: 9004-34-6, microcrystalline, powder, ca. 20 μm, Sigma Aldrich) and xylan (CAS: 9014-63-5, xylan from beechwood, Sigma Aldrich) were taken as references. Their Raman spectra were acquired in the range of 580-3062 cm^−1^ under the same acquirement conditions as the sample.

### Data analysis

The system framework diagram of this study is shown in Fig. 1. After Raman spectrum acquisition, the data preprocess was adapted to eliminate disturbances from random noise and fluorescent backgrounds. Then the processed spectra of the average Raman response of various tissues and the map scanning of transections were taken for statistical analysis, respectively. In terms of classification, qualitative and quantitative classifications of untreated and alkali-treated straw based on spot scanning spectra were developed, and fingerprint bands for classification were also obtained. Moreover, spectral chemical imaging was realized by spectral unmixing analysis of fully constrained least-squares (FCLS) with a full-range spectral constraint of lignocellulosic standards; therefore, a visualization and quantification of subcellular lignocellulose from FCLS was obtained and compared to the single-band spectral images and Safranine O–Fast Green staining image. Finally, these qualitative, quantitative, and location analyses were integrated with the results of traditional methods including the electron microscope image, lignocellulosic content, and biogas production, revealing the mechanism of alkali pretreatment increasing the methane yield of rice straw in anaerobic digestion. All procedures were implemented in Matlab R2013b (The Math Works, Natick, MA, USA).

#### Noise reduction

The non-sample region (background) had a negative effect on the subsequent characterization of the sample images (foreground) [12]. As there were obvious spectral differences between the samples tissues (rice straw) and the background (spurr resin), and as such a threshold segmentation method was used in this study to remove the background.

Cosmic ray spikes often occur in Raman spectra, which seriously distort the Raman spectra of the substances to be measured, and affect the acquisition of attribute information of the measured samples, need to be removed in advance. Adaptive iteratively reweighted penalized least-squares (airPLS) can be used to eliminate high-frequency noise, cosmic rays and correct the baseline background [26, 27]. AirPLS was used to eliminate cosmic rays in this paper.

To improve the signal-to-noise ratio, principal component analysis (PCA), which can extract main information through projecting data into several orthogonal variable spaces with the greatest extent of data variance, was carried out for noise elimination [28]. The Raman spectra were firstly decomposed into its principal components, and then de-noising spectra were reconstructed using the first three principal components with more than a 99.99% explained variation of the original spectra.

Normalization is often used to correct spectral changes caused by minute optical path differences. Area normalization was used in this study. The principle is as follows:

1 $$x_{ij}^{\prime} = {\raise0.7ex\hbox{${x_{ij} }$} \!\mathord{\left/ {\vphantom {{x_{ij} } {\mathop \sum \nolimits_{j} x_{ij} }}}\right.\kern-0pt} \!\lower0.7ex\hbox{${\mathop \sum \nolimits_{j} x_{ij} }$}} ,$$

where *x*_*ij*_ represents the spectral intensity value in pixels *i* and band *j*, $x_{ij}^{\prime}$ represents the spectral intensity after area normalization.

#### Feature extraction

**Fig. 2:**
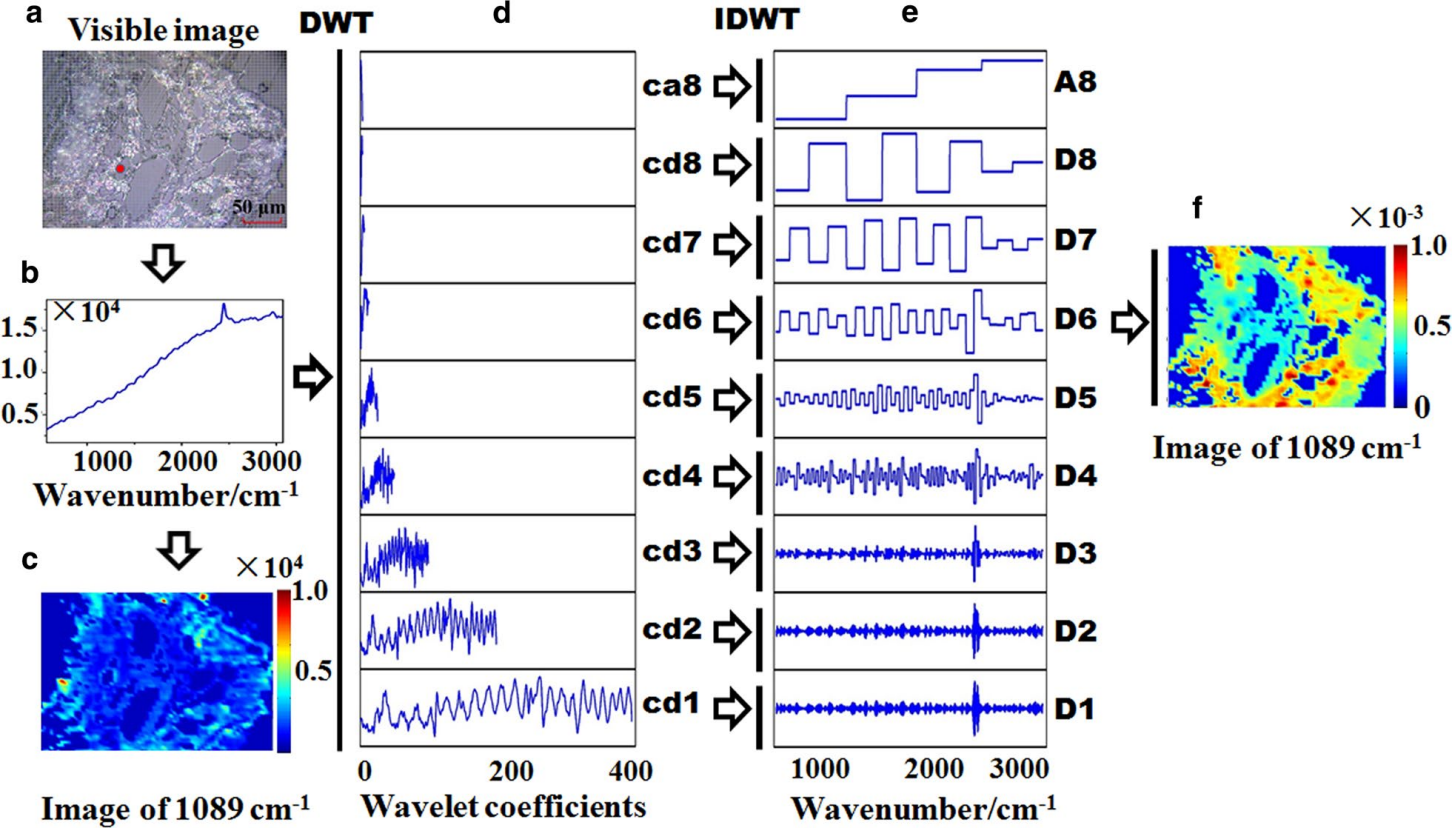
Extraction of spectral characteristic with wavelet transform

Wavelet transform (WT) is a powerful feature extraction algorithm that deconstructs the signal (spectrum) into the sum of its functions (wavelet) with different spatial and frequency properties [29–31]. By deconstructing the spectra data of different scales and frequencies, the inherent structure and characteristic information of spectral data can be discovered [32, 33]. The data can be accurately constructed by wavelet analysis with relatively few components [34]. Among them, discrete wavelet transform (DWT) is widely used [35]. A flowchart of the process of extracting characteristic information from spectra by wavelet transform is shown in Fig. 2. Taking the spectrum of the red point in the map scan as an example (Fig. 2a), the profile of this spectrum is shown in Fig. 2b. Firstly, the spectrum was deconstructed into nine sets of wavelet coefficients at level 8 by discrete wavelet transform (DWT) (Fig. 2d). Then inverse discrete wavelet transform (IDWT) was used to reconstruct the spectral signal from the nine sets of wavelet coefficients respectively (Fig. 2e). Furthermore, spectral image of 1089 cm^−1^ was produced, pixel by pixel, based on the reconstructed spectral information based on D6 (Fig. 2e), while spectral image of 1089 cm^−1^ based on the raw spectra also developed as shown in Fig. 2c. In this paper, WT was used to extract features and eliminate the influence of high-frequency noise and low-frequency fluorescence interference.

#### Classification

Principal component analysis (PCA) was adopted to cluster the analysis of samples before and after alkali treatment. It applies a linear transformation to decompose spectral data into several principal components (PCs), which are not correlated [36, 37]. The first two PCs are utilized to analyze the common features among samples and their grouping [38].

Two supervised classification techniques, including linear discriminant analysis (LDA) and K-nearest neighbor (KNN) were used for quantitative differentiation of samples before and after alkali treatment. For the specific principle, previous literature can be consulted [39, 40].

#### Spectral unmixing with fully constrained least-squares (FCLS)

Linear spectral mixture analysis (LSMA) is a widely used technique for estimating the abundance fractions of constituents present in multi-spectral/hyper-spectral image pixels [41–44]. The principle is as follows:

2 $$D_{{\,\left( {x\, \times \,y} \right)\, \times \,L}} = C\,_{{\left( {x\, \times \,y} \right)\, \times \,k}} P_{k\, \times \,L}^{\text{T}} + R_{{\left( {x\, \times \,y\, \times \,L} \right)}},$$

where *D* is a (*x* × *y*)×* L* spectrum matrix of a spatially unfolded Raman micro-spectrum image (Fig. 1), *x*, *y* are the number of pixels of the vertical and horizontal axis of the map scanning area, and *L* is the number of spectral bands. *P*^T^ is a *k *×* L* matrix of *k* pure component spectra. Here, *D* and *P*^*T*^ were adjusted to [0,1] by maximum normalization. For the *k* pure component spectra, *C* is an (*x *×* y*)×* k* matrix of mixing coefficients (concentrations), and *R* is the residual matrix.

The unknown abundance fractions are calculated by solving the inverse solution of the linear mixing model specified by (2), so as to complete the tasks of material discrimination, detection, classification, etc. [42].

Heinz and Chang [42] proposed the fully constrained least-squares (FCLS) method as an estimator based on LSMA to produce an accurate amount of constituent abundance. The FCLS method imposes two constraints on the linear mixture model used in LSMA, which are the abundance sum-to-one constraint and the abundance non-negativity constraint [41–43, 45], expressed as follows:

3 $$\mathop \sum \limits_{j = 1}^{k} C_{j} = 1,$$

4 $$C_{j} \ge 0\,\left( {1 \le j \le k} \right).$$

The abundance (concentration) is corrected by the measured percentage content of the $k$ components in the target object.

5 $$\left( {C_{j} } \right)_{\text{cal}} = C_{j} \times W ,$$

where *C*_*j*_ is the concentration of all *k* components calculated by the FCLS method. *C*_*j*__cal_ is calibrated according to the sum of the concentration (*W*) of all the *k* components based on laboratory analysis.

## Results and discussion

### Raman spectral unsupervised clustering of rice straw before and after alkali treatment

**Fig. 3:**
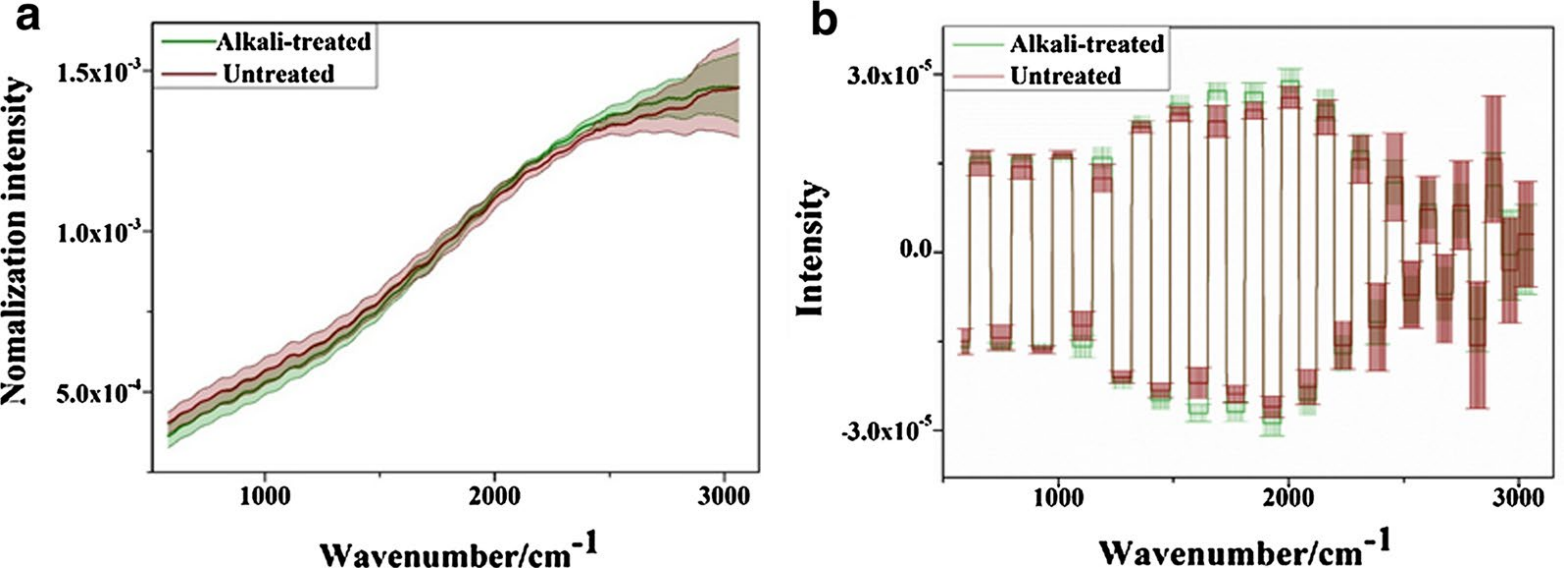
Raman spectral response of untreated and alkali-treated rice straw. **a** Noise reduction. **b** Noise reduction + WT

**Fig. 4:**
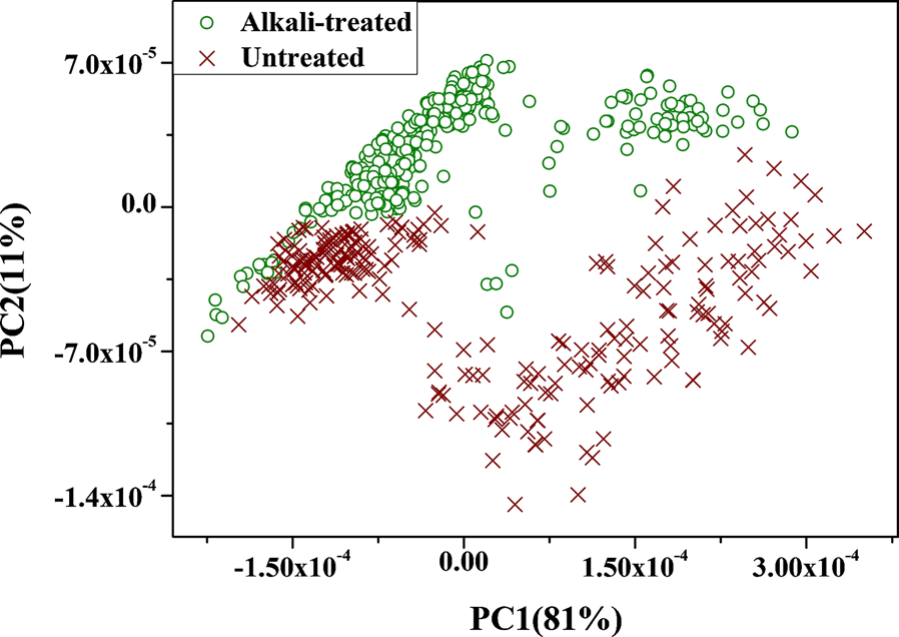
PCA scores plot of Raman spectra from untreated and alkali-treated rice straw

The Raman spectra of untreated and alkali-treated samples after noise reduction are shown in Fig. 3a, it was found that there were similar spectral response characteristics, and many overlapping samples of the two types. It is worth noting that there were strong fluorescent backgrounds, which tended to increase with the increase of wave number. These fluorescent backgrounds may be caused by chromophores (pigment) in plant tissues [21, 46]. WT treatment was carried out to eliminate the fluorescence background, and reconstructed spectral information based on D6 is shown in Fig. 3b. It worth noting that the fluorescent background which increases along with the increase of wave number was greatly reduced by WT treatment, especially in the region of high wavenumbers. Although the fluorescence background was mostly eliminated by WT, the spectra were still overlapped. In order to capture the Raman spectral characteristics of untreated and alkali-treated rice straw tissues, their Raman spectra after WT pretreatment were analyzed by PCA in the whole bands (580–3062 cm^−1^). It can be found that the cumulative variation of the first two principal components reached 92%, and the projection of samples in the two principal component spaces is shown in Fig. 4. The abscissa indicated that the first principal component (PC1) score of the sample and the Y-coordinate represents the second principal component (PC2) score value of the sample.

As is evident in Fig. 4, most of the untreated samples can be effectively distinguished from alkali-treated samples in the PC1 and PC2 space, except for a few samples that overlapped. This means that Raman spectroscopy could capture the difference of untreated and alkali-treated rice straw samples. Because the shift, number, and intensity of peaks in Raman spectrum were directly related to the molecular vibration or the rotational energy level of the sample, it was expected to reveal the changes of molecular composition and structure in rice straw before and after alkali treatment by analyzing the Raman spectral difference.

**Fig. 5:**
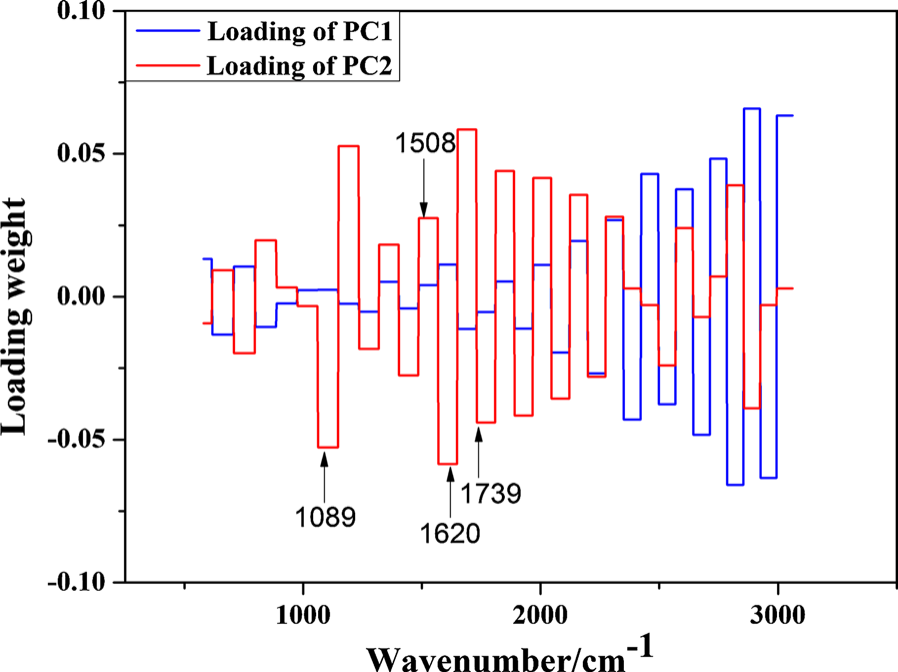
Loading weight plot of the first two principal components

The relationship between principal component and Raman spectrum data can be reflected by loading weight, so the analysis of loading weight of principal component will help to reveal the differences of spectral characteristics and corresponding components between rice straw before and after alkali treatment. The loading weight plot of the first two principal components is shown in Fig. 5. The peak with large loading weight is the main factor affecting the principal component. In this paper, the bands whose absolute value of loading weight is greater than 0.025 were analyzed. Among the analyzed bands, the Raman bands of 1089, 1508, 1620 and 1739 cm^−1^ are all related to lignocellulose. In detail, the Raman band of 1089 cm^−1^ can be assigned to C–O–C and C–C ring vibrations in hemicellulose [47], and C–O and C–C stretching in cellulose [48], the peaks of 1620 cm^−1^ are associated with the ring conjugated C=C str. of coniferaldehyde [49, 50], the intensity of Raman band near 1508 cm^−1^ is related to asymmetric stretch vibration of benzene ring in lignin [51], 1739 cm^−1^ band region is associated with C=O stretching vibration in hemicellulose [52]. In conclusion, lignocellulose may be the key to the differences of spectral characteristics and corresponding components between rice straw before and after alkali treatment.

### Quantitative classifier and chemical micro-imaging with fingerprint bands

#### Quantitative classifier

In order to quantitatively reveal the Raman spectral difference between untreated and alkali-treated rice straw samples, quantitative classifiers were developed based on the LDA and KNN algorithms. The untreated and alkali-treated samples were randomly divided into training and prediction sets in a ratio of 3:1. The preprocessed spectral data and nine groups of single-branch reconstruction signals from 1-D wavelet decomposition coefficients at level 8 were respectively taken as input to establish the classifier. Except of full range, the optimal combination of bands was also selected for classification. In terms of classification algorithm, it was found that KNN almost outperformed LDA in classification accuracy and stability, as is shown in Table 1.

**Table 1:** Classification accuracy of quantitative classifier

Algorithm	Input feature	Raw	A8	D1	D2	D3	D4	D5	D6	D7	D8
LDA	Full-range spectra	80.56	47.92	75.00	79.17	84.72	76.39	88.19	79.86	78.47	57.64
	Two bands (1620 + 1089 cm^−1^)	87.22	90.28	85.42	84.06	85.42	84.72	86.81	88.89	88.89	90.28
KNN	Full-range spectra	79.86	84.72	84.03	85.42	86.81	84.03	93.75	95.14	96.53	87.5
	Two bands (1620 + 1089 cm^−1^)	82.64	86.11	85.42	86.11	85.42	83.33	88.89	95.14	93.75	90.28

Interestingly, the classifiers based on the wavelet single-branch reconstruction signals frequently obtained a higher accuracy than those based on the raw spectral data when the decomposition level was set from 5 to 7, indicating that Raman spectra suffered serious interference from noise, although high-frequency cosmic rays and random noise had been reduced by the airPLS and PCA, respectively. Furthermore, as the level increased from 1 to 5, the classification accuracy basically increased. This may be owed to the fact that the low-frequency fluorescence signal which was always accompanying and interfering with Raman spectra of plant tissue was suppressed by the wavelet single-branch reconstruction at a higher level. The classifier based on single-branch reconstruction signals from 1-D wavelet decomposition coefficients at level 6 (D6) also obtained a high and stable accuracy, whether in full-range spectra or feature bands modeling. Hence, D6 were taken as optimal features when separated from noise to replace the spectral data for further analysis. In combination with the reconstruction schematic diagram of WT in Fig. 2, it could be concluded that D6 extracts the characteristic information of the raw spectrum through eliminating high-frequency random noise and low-frequency fluorescent backgrounds [32, 53, 54].

**Fig. 6:**
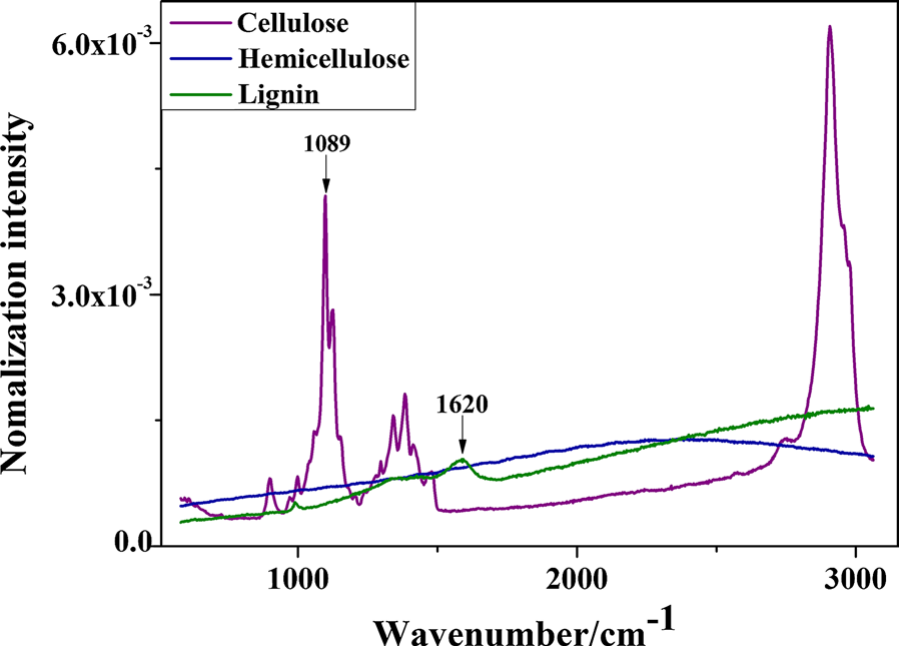
Raman spectra of lignocellulosic standards

It could be concluded that the classification accuracy of the KNN classifier of D6 with two bands of 1620 and 1089 cm^−1^ was the same as that of the full-range spectrum, reaching 95.14%. This indicates that these two bands of 1620 and 1089 cm^−1^ were the fingerprint characteristics for classification of untreated and alkali-treated rice straw samples. To illustrate the assignment of these peaks, the Raman spectra of lignocellulosic reference standards were acquired, as is shown in Fig. 6. It could be concluded that there were obvious peaks (including 1089 cm^−1^) of cellulose, which were almost free from fluorescence interference, while a peak of lignin appeared at 1620 cm^−1^. Furthermore, hemicellulose and lignin were disturbed by fluorescence, especially for hemicellulose without obvious peaks, indicating that the Raman spectra of hemicellulose and lignin are accompanied by strong fluorescence. This may be the reason why the Raman spectra of rice straw samples show a large amount of fluorescent backgrounds, as is shown in Fig. 3a.

The Raman bands of 1089 and 1620 cm^−1^ were identified as a lignocellulose indicator, indicating that the chemical compounds of cellulose, hemicellulose, and lignin played an important role in the differentiation of untreated and alkali-treated rice straw. This conclusion was consistent with the macroscopic measurement content of lignocellulose from conventional wet chemistry methods (Table 2), which showed that hemicellulose, lignin, and cellulose were reduced by 53%, 25% and 13%, respectively, by alkali treatment. The above results showed that the Raman spectral classification of the rice straw before and after alkali treatment was realized by capturing the characteristic spectral information of lignocellulose.

**Table 2:** Main composition contents of untreated and alkali-treated rice straw (%, dry base, d.b.)

	Cellulose (sd)	Hemicellulose (sd)	Lignin (sd)	CHL	ΔCHL	Ash
Untreated	39.5 (3.0)	33.2 (0.5)	4.5 (0)	77.2	0	2.6(0.2)
Alkali-treated	34.4 (0.8)	15.6 (1.1)	3.4 (0.3)	53.4	15.8	2.0(0.3)

*CHL* total contents of cellulose, hemicellulose, and lignin, Δ*CHL* variation of CHL, *sd* standard deviation

#### Chemical micro-imaging with classification fingerprint bands

**Fig. 7:**
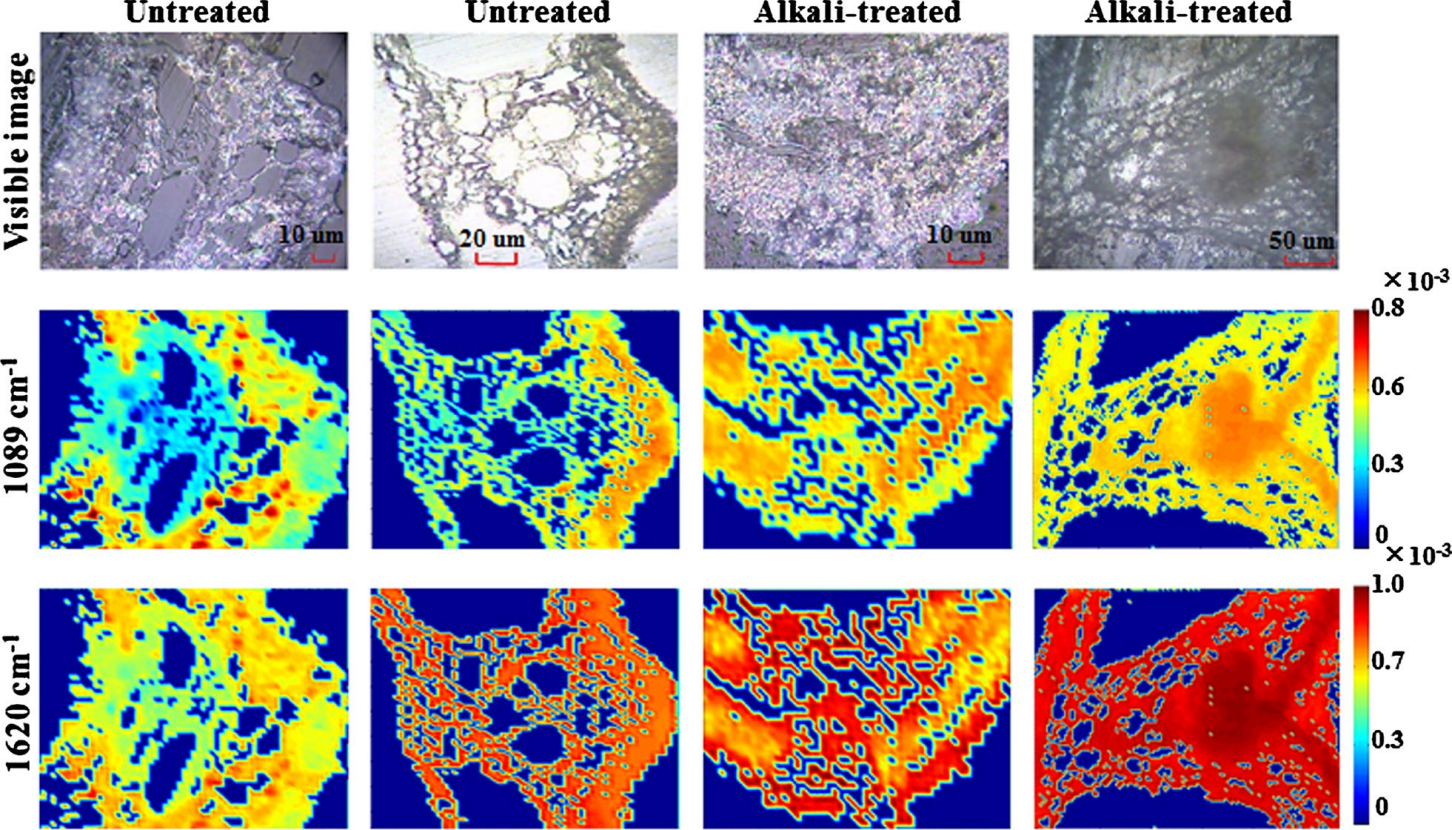
Raman images at characteristic bands of transverse sections of untreated and alkali-treated rice straw

As these two bands of 1620 and 1089 cm^−1^ were the fingerprint characteristics for classification, the single-intensity spectral images based on the two bands of 1620 and 1089 cm^−1^ may reveal the intrinsic difference between untreated and alkali-treated samples. The spectral images are shown in Fig. 7. It was found that there are highly similar subcellular spatial distributions between spectral images of 1089 and 1620 cm^−1^; however, these two bands were obvious different in their spectral intensities. Because these two peaks belonged to polysaccharide and lignin respectively, their spatial distribution should be different in theory [20]. The illogicality of the spectral imaging (with 1089 or 1620 cm^−1^) was probably due to the interference of strong background fluorescence, which submerged the spectral attributes of the two bands (1089 and 1620 cm^−1^), and resulted in the high similarity of the single-band spectral imaging. As highly similar images of cellulose and lignin had also been previously reported [55], it could be concluded that fluorescence interference is a common phenomenon in Raman spectral imaging. Although various pretreatment methods including airPLS, PCA, normalization, and WT had been adopted, the fluorescence interference had not been completely eliminated. Thus, Raman single-band spectral imaging was inappropriate for mapping chemical distributions of constituents in plant tissues with a high fluorescence background.

Notably, alkali treatment reduced hemicellulose, lignin, and cellulose contents by 53%, 25%, and 13%, respectively, as is shown in Table 2; however, the degradation effect of alkali treatment on the subcellular spatial distribution of hemicellulose, cellulose, and lignin could not be illustrated in the spectral images of 1089 and 1620 cm^−1^. As shown in Fig. 7, the spectral images of 1089 and 1620 cm^−1^ for alkali-treated samples exhibited a higher intensity than that of untreated samples. If the Raman spectral intensity at 1089 and 1620 cm^−1^ were significantly linearly related to the contents of polysaccharide (hemicellulose and cellulose) and lignin respectively, it showed that the content of polysaccharide and lignin did not decrease, but increased after alkali treatment. This result contradicts the macroscopic lignocellulosic content through wet chemical measurement method. The reason for the inconsistency between the single-band spectral imaging (1089 and 1620 cm^−1^) and the concentration of macro-detection may be that the single-band spectra were still disturbed by fluorescent backgrounds, which destroyed the linear relationship between the concentration of the compound and the Raman spectral intensity at the well-defined band. Furthermore, chemical treatment (including acid and alkali treatments) often results in spectral band broadening or shifting. Thus, Raman single-band spectral imaging can’t expose compounds distribution of plant tissue interfered by fluorescence background, a more reliable method of chemical imaging of lignocellulosic distribution was urgently needed.

### Chemical imaging of untreated and alkali-treated rice straw based on the FCLS

**Fig. 8:**
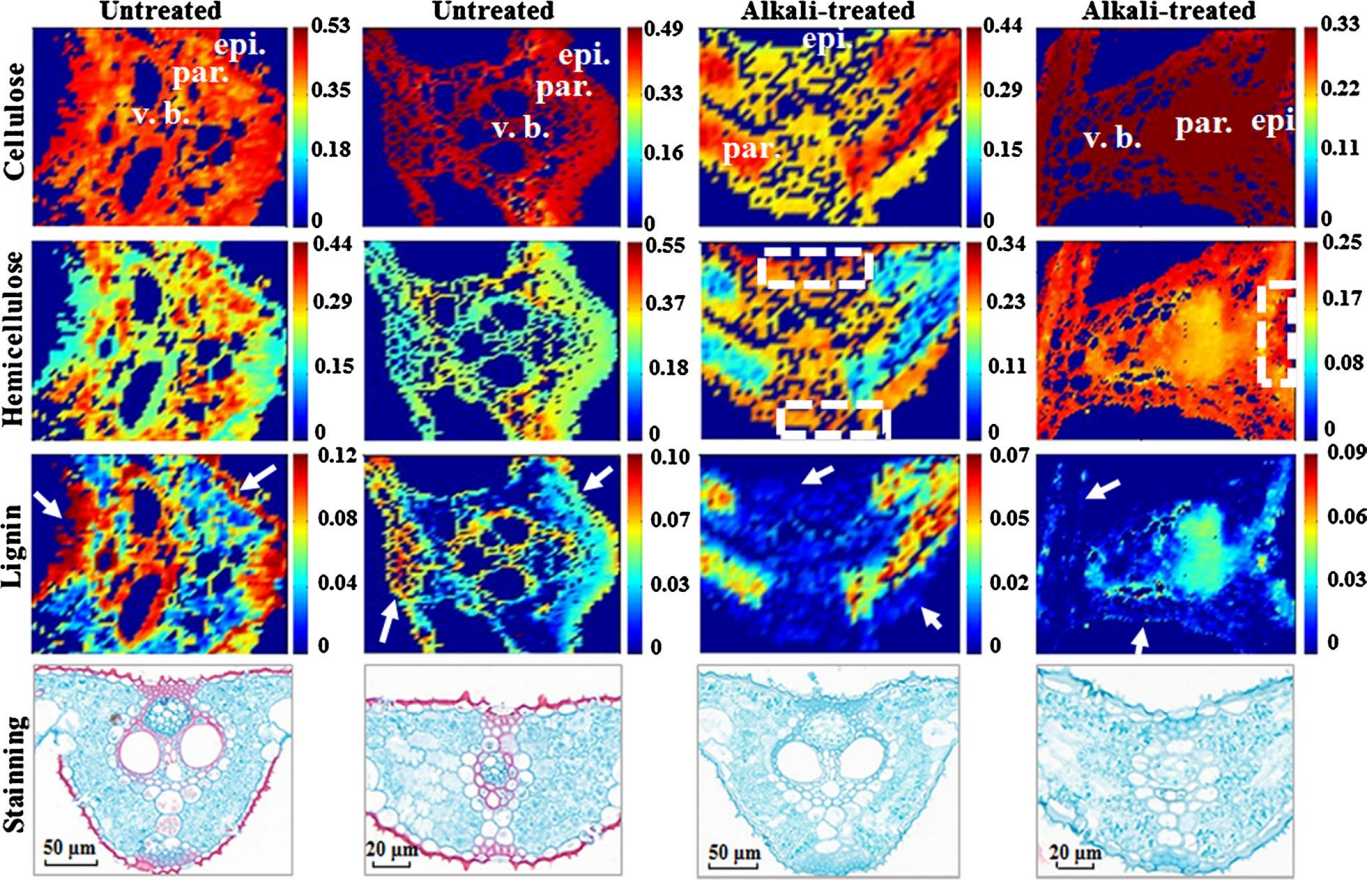
Chemical imaging of lignocellulose by spectral unmixing analysis of the FCLS. (in the fourth row, the sections were stained with Safranin O–Fast Green. Safranin O stained lignin red, Fast Green stained cellulose green). *vb* vascular bundle, *par* parenchyma, *epi* epidermis

**Fig. 9:**
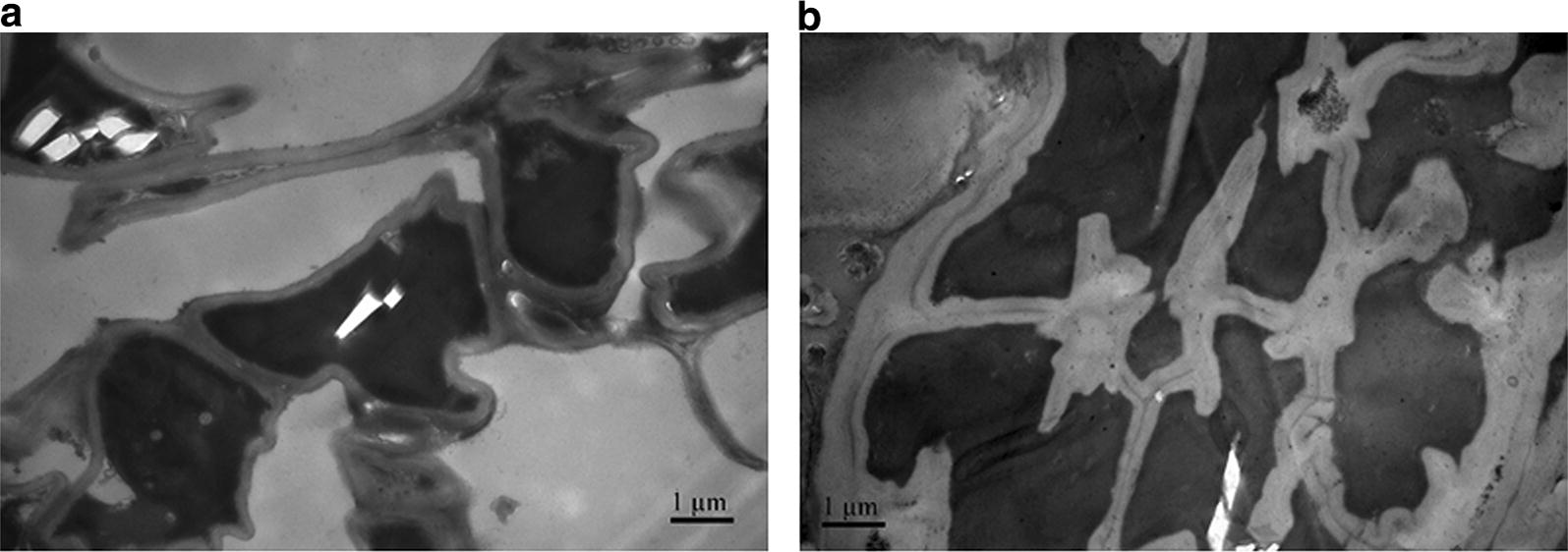
Transmission electron microscope images of rice straw tissues. **a** Untreated rice straw tissue. **b** Alkali-treated rice straw tissues

To obtain more reliable images of subcellular lignocellulosic distribution, spectral unmixing of the FCLS was adopted based on the reference spectra of lignocellulosic standards (as shown in Fig. 6), which was used as a spectral full-range constraint to resolve the lignocellulosic concentration at each sampling point in micro-Raman mapping. Chemical images of lignocellulose were obtained by the FCLS as shown in Fig. 8. It can be found that unpretreated materials showed a highly smooth, dense and uniformly arranged fibrous structure. However, NaOH pretreatment leaded to serious damage to the surface structure, forming a new pattern with rough surface, more cracks and voids, and large surface area (the position shown in the dotted white box in Fig. 8), which was consistent with the scanning electron micrographs in previous research by Amir [55]. With regard to the subcellular level by electron microscopy (Fig. 9), it can be found that the outlines of cells in the untreated tissue were clear, and the cell wall had a uniform thickness and good integrity. While, there was swelling and thickening of cell walls in the alkali-treated rice straw tissues, and the cell walls of many cells were broken to some extent. Especially, tissue expansion and microstructure fracture occurred in the alkali-treated samples compared with untreated ones. These phenomenon may be due to the alkali treatment loosening the dense cross-linking structure of lignocellulose [56]. In addition, it was found that the subcellular cellulose, hemicellulose, and lignin could be visualized and quantified, which was highly consistent with the macroscopic concentration measurement results (as shown in Table 2) and the Safranin O–Fast Green staining images (as shown in the fourth line of Fig. 8). In particular, the chemical image of lignin (in the third line of Fig. 8) clearly showed a severe degradation of lignin in epidermal cells by alkali treatment (the position indicated by the white arrow in the third line of Fig. 8), which was also confirmed by Safranin O–Fast Green staining images. In Amir’s study [55], author speculated that NaOH pretreatment greatly reduced the proportion of lignin in the surface structure of biomass [57], effectively opened up the rigid structure of Cogongrass, and facilitated the direct contact between enzyme and cellulose [58], and this is the first time that this hypothesis has been confirmed by lignin imaging analysis at subcellular level in this study. At the same time, the changes of content and distribution of cellulose and hemicellulose, which could not be detected by Safranin–Fast Green staining, could also be detected in the chemical images in this study (as shown in Fig. 8). It can be found that for untreated samples, cellulose was well and densely distributed, and hemicellulose was concentrated in parenchyma with punctate granules. Lignin was mainly distributed in epidermal cells, xylem and sclerenchyma [12]. For alkali-treated samples, there was a dramatic decline in the contents of lignocellulose (cellulose, hemicellulose, and lignin) after alkali treatment, and the high orderly spatial structures of lignocellulose were also destroyed. It is noteworthy that most of the lignin in epidermal cells, xylem and sclerenchyma is degraded, especially in the epidermal cells and vascular bundle cavities, as there’s a lot of contact with sodium hydroxide solution in these areas. Therefore, different types of cells and their spatial distribution differences in tissue lead to significant spatial differences in the effect of alkali treatment. It is worth noting that alkali treatment resulted in a centralized distribution of lignin in the transection, with less near the upper and lower epidermis. This may be due to the hydrolysis of lignin being greatly dependent on access to the alkali solution; therefore, high accessibility between the epidermis and alkali solution lead to a significant degradation of lignin, as well as to less lignin degradation in the middle of cross sections along with less accessibility to the alkali solution. This result was consistent with former research [59], in which the lignin distribution change in fiber cells of *Eucalyptus* was illustrated by Raman images with the integration of band intensity (1547–1707 cm^−1^), however, with the exception of fiber cells, other types of cells had not been studied. Furthermore, a distribution change of polysaccharides, the core components of lignocellulosic biomass transformation, had also not been studied [59].

**Fig. 10:**
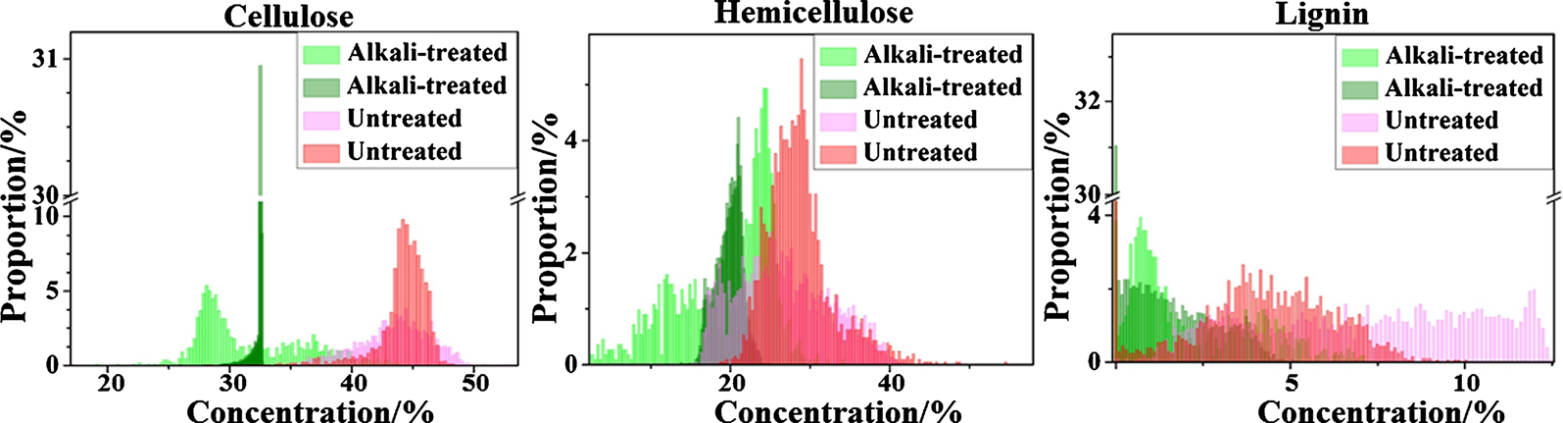
Lignocellulosic content histogram of Raman chemical images based on spectral unmixing of FCLS

From the histogram of lignocellulose contents of each pixel in chemical images (Fig. 8) before and after alkali treatment shown in Fig. 10, it was found that the distribution of cellulose, hemicellulose, and lignin tend to be in a lower concentration range after alkali treatment. In other words, alkali treatment caused an obvious decline in the concentration of cellulose, hemicellulose, and lignin in microscopic transection, which was highly consistent with macroscopic lignocellulosic concentrations measured by wet chemical measurement (as shown in Table 2).

**Fig. 11:**
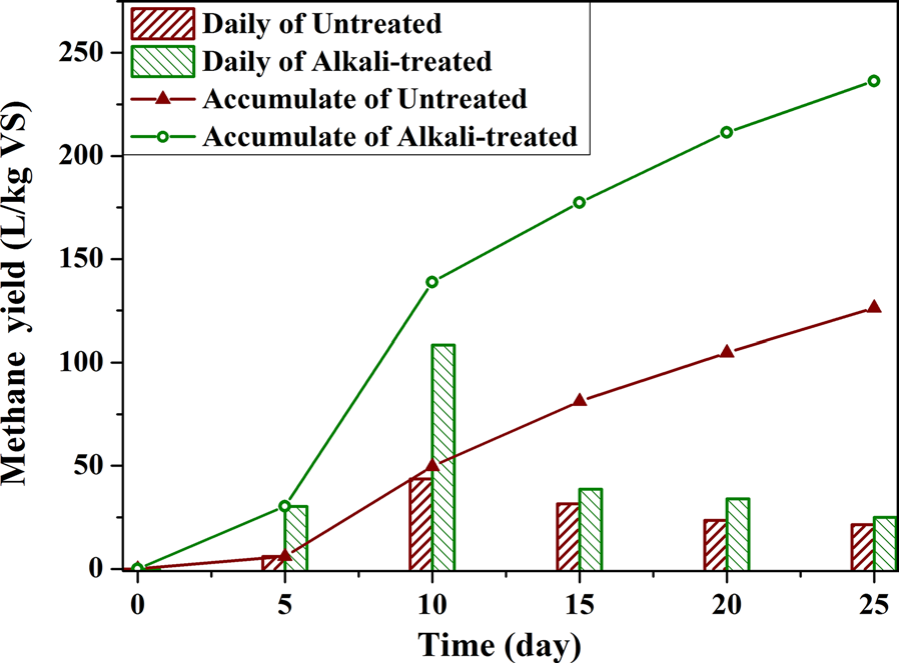
Biogas production per kilogram vs of substrates

In terms of the gas production as shown in Fig. 11, alkali treatment accelerates methane production speed in the first 10 days. The methane yield of the alkali-treated group was about three times that of the untreated group in the first 5 days. The results indicated that alkali treatment could solve the problem of slow start-up of anaerobic fermentation gas production. Moonkyung et al. found that alkali pretreatment improved the decomposition ability of lignocellulose and the production rate of methane [60]. This may be due to the fact that alkali treatment severely degraded the high concentration of lignin in the epidermis and increased the accessibility of the alkali solution and microorganisms to polysaccharides in the inner cells of tissues (as shown in Fig. 8). Furthermore, the methane yield increased by 87.1% compared to the untreated group (from 126.30 to 236.35, as shown in Fig. 11), which was consistent with previous research [61, 62], while the direct degradation of lignocellulose by alkali treatment was about 15.8%, as is shown in Table 2.

Integrating the macro-measurement with the micro-chemical images indicated that the contribution of alkali treatment to gas production can be divided into two parts: the one is to change the chemical properties of rice straw by direct degradation and dissolution of lignocellulose components, another is to remove the structural barriers of lignin in epidermal cells, xylem and sclerenchyma. And the latter plays a greater role in promoting methane gas production.

The high consistency of the results of chemical imaging of subcellular lignocellulose by means of the FCLS strategy, macroscopic concentration measurement, and chemical dyeing indicated that the spectral unmixing of FCLS with the full-range spectra constraint of the reference standard was a reliable and effective Raman chemical imaging method. The highlight of this strategy was getting rid of the traditional single-band chemical imaging method based on the spectrum, with fluorescence disturbance eliminated thoroughly, which was difficult to realize without a clear understanding of the fluorescence orientation of biological tissue. Instead, with the FCLS strategy, fluorescent backgrounds carried by both standard references and mapping samples were also taken as part of the clues for the decomposition of the micro-Raman mapping spectra with fluorescence signals. Therefore, fluorescent backgrounds, which were also frequently accompanied with Raman spectra, were combined with Raman spectroscopy to promote chemical imaging analysis.

## Conclusion

It could be concluded that it is feasible to nondestructively detect changes of subcellular hemicellulose, lignin, and cellulose in rice straw tissue induced by alkali treatment based on this Raman chemical imaging method. The label-free chemical imaging strategy of integrating FCLS with the full-range spectra constraint of the reference standard especially provided an effective approach to visualize and quantify the subcellular distributions of lignocellulose, which was highly consistent with Safranin O–Fast Green staining images and macroscopic concentrations of lignocellulose from wet chemical measurements. The biggest advantage of this strategy is that the full-range of spectral signals, including the partial fluorescent backgrounds, were effectively integrated for chemical imaging analysis, so it was more robust and reliable than traditional single-band spectral imaging. Moreover, the percentage content of subcellular lignocellulose could be plotted based on the spectral unmixing analysis with spectra of standards as global constraints.

The label-free chemical imaging revealed the temporal and spatial distribution characteristics of lignocellulose of dozens of cells induced by alkali treatment at the subcellular level. There was a dramatic decline in the contents of hemicellulose, cellulose, and lignin in all cells of the transection after alkali treatment, especially in epidermal tissue cells and adjacent areas. These results indicated that the alkali treatment increased the methane production rate, mainly due to its effective elimination of structural recalcitrance of biomass by breaking down high lignin concentrations in the epidermis to increase the accessibility of alkali and microorganisms to polysaccharide in the inner cells of the transection.

A qualitative, quantitative, and location analysis of subcellular lignocellulose of rice straw under alkali treatments were established in this paper by combined confocal micro-Raman spectroscopy with chemometrics, which provided a new approach for deep understanding the mechanism of alkali treatment promoting gas production and accelerating the initial gas production rate. The further development of such an approach could promote efficient utilization of biomass.

## Data Availability

Part applicable on reasonable request.

## Abbreviations

airPLS: adaptive iteratively reweighted penalized least-squares
CARS: coherent anti-Stokes Raman scattering
CHL: total contents of cellulose, hemicellulose, and lignin
ΔCHL: variation of CHL
db: dry base
DWT: discrete wavelet transform
D6: 1-D wavelet decomposition coefficients at level 6
Epi: epidermis
FCLS: fully constrained least-squares
IDWT: inverse discrete wavelet transform
KNN: K-nearest neighbor
LDA: linear discriminant analysis
LSMA: linear spectral mixture analysis
Par: parenchyma
PCA: principal component analysis
PCs: principal components
PC1: first principal component
PC2: second principal component
sd: standard deviation
SRS: stimulated Raman scattering
TS: total solid content
vb: vascular bundle
VS: volatile solids content
wb: wet base
WT: wavelet transform
